# ‘Not taking medications and taking medication, it was the same thing:’ perspectives of antiretroviral therapy among people hospitalised with advanced HIV disease

**DOI:** 10.1186/s12879-024-09729-8

**Published:** 2024-08-13

**Authors:** Marian Loveday, Sindisiwe Hlangu, Pariva Manickchund, Thiloshini Govender, Jennifer Furin

**Affiliations:** 1 HIV and Other Infectious Diseases Research Unit (HIDRU), South African Medical Research Council, 491 Peter Mokaba Ridge Road, Overport, Durban, KwaZulu-Natal South Africa; 2 Centre for Health Systems Research & Development, University of the Free State, Bloemfontein, South Africa; 3 CAPRISA-MRC HIV-TB Pathogenesis and Treatment Research Unit, Doris Duke Medical Research Institute, University of KwaZulu-Natal, Durban, South Africa; 4 Internal Medicine, King Edward VIII Hospital, KwaZulu-Natal Department of Health, University of KwaZulu-Natal, Durban, South Africa; 5 King Dinuzulu Hospital Complex, KwaZulu-Natal Department of Health, Durban, South Africa; 6 Department of Global Health and Social Medicine, Harvard Medical School, Boston, USA; 7 Division of Infectious Diseases and HIV Medicine, Case Western Reserve University and University Hospitals Cleveland Medical Center, Cleveland, OH USA

**Keywords:** South Africa, Advanced HIV disease, Retention in care, Tuberculosis

## Abstract

**Background:**

Despite HIV's evolution to a chronic disease, the burden of advanced HIV disease (AHD, defined as a CD4 count of < 200 cells/uL or WHO clinical Stage 3 or 4 disease), remains high among People Living with HIV (PLHIV) who have previously been prescribed antiretroviral therapy (ART). As little is known about the experiences of patients hospitalised with AHD, this study sought to discern social forces driving hospitalisation with AHD. Understanding such forces could inform strategies to reduce HIV-related morbidity and mortality.

**Methods:**

We conducted a qualitative study with patients hospitalised with AHD who had a history of poor adherence. Semi-structured interviews were conducted between October 1 and November 30, 2023. The Patient Health Engagement and socio-ecological theoretical models were used to guide a thematic analysis of interview transcripts.

**Results:**

Twenty individuals participated in the research. Most reported repeated periods of disengagement with HIV services. The major themes identified as driving disengagement included: 1) feeling physically well; 2) life circumstances and relationships; and 3) health system factors, such as clinic staff attitudes and a perceived lack of flexible care. Re-engagement with care was often driven by new physical symptoms but was mediated through life circumstances/relationships and aspects of the health care system.

**Conclusions:**

Current practices fail to address the challenges to lifelong engagement in HIV care. A bold strategy for holistic care which involves people living with advanced HIV as active members of the health care team (i.e. ‘PLHIV as Partners’), could contribute to ensuring health care services are compatible with their lives, reducing periods of disengagement from care.

**Supplementary Information:**

The online version contains supplementary material available at 10.1186/s12879-024-09729-8.

## Background

Advanced HIV disease (AHD)—defined as a CD4 count of less than 200 cells/uL and/or World Health Organization Stage 3 or 4 disease—is a leading cause of global morbidity and mortality [1]. AHD may be related either to delays in initial HIV diagnosis or failure of highly active antiretroviral therapy (ART) [2]. ART may be ineffective for biological reasons, including drug resistance, inadequate drug absorption, or drug-drug interactions that lead to sub-optimal drug levels [3]. There are also a range of social factors that can contribute to the ineffectiveness of ART, including challenges with medication adherence or disengagement from HIV care [4].

The 95–95-95 HIV care cascade—which aims to have 95% of people living with HIV know their status, 95% of those knowing their status on treatment, and 95% of those on treatment with suppressed viral loads [5], depicts HIV care as linear and unidirectional starting at the time of diagnosis and ending with treatment discontinuation or death. This cascade simplifies the complex treatment journey of People Living with HIV (PLHIV) which includes periods of engagement, disengagement and re-engagement, in what has been described as a cyclical cascade [6, 7]. “Disengagement” in health care is defined as a period or periods of interruption in regularly scheduled services [8]. Because ART is a life-long therapy, people living with HIV must be engaged in care for prolonged periods of time [9]. A systematic review estimated that 25% of PLHIV on ART interrupt their treatment at some stage [10, 11]. Disengagement is usually associated with interruption of ART, which can result in AHD if the period of disengagement is prolonged [12].

In South Africa, studies have shown that AHD is associated with as many as 60% of hospital admissions and that among those hospitalised with AHD, 64.3% were prescribed ART [13]. Hospitalised individuals with HIV have high rates of mortality, with studies showing that upwards of 25% die while in the hospital [14]. An additional 25% of people hospitalised with AHD die within one year of hospitalisation [15]. TB is the most common cause of hospital admission and death in PLHIV and accounts for up to a third of HIV-related deaths [16, 17], and inpatient mortality [18].

Understanding the treatment journeys of individuals hospitalised with AHD is important in reducing morbidity and mortality from HIV, as these individuals’ experiences may be especially informative for identifying “where things went wrong” [19]. While there have been qualitative explorations of why people diagnosed with HIV fail to initiate treatment [20], there are only limited studies exploring the treatment journeys of people who are hospitalized with AHD. Studies that have been done were limited geographically but found that stigma, religious beliefs, and economic factors were important drivers of disengagement from HIV care [21]. To expand the knowledge base among this vulnerable population, we undertook an exploratory, qualitative study to describe the treatment experiences of people hospitalised with advanced HIV in KwaZulu-Natal, South Africa.

## Methods

### Study design

Given the limited research on treatment journeys of people hospitalised with AHD, we performed an exploratory, mixed-methods study in this population. As part of the study, open-ended, semi-structured qualitative interviews were conducted. We report the qualitative data here.

### Study context

The study was conducted at King Edward VIII and King Dinizulu Hospitals in KwaZulu-Natal. King Edward Hospital is a busy tertiary level hospital with 852 beds, to which many patients with AHD in the Durban metro area are admitted. King Dinuzulu is a regional referral hospital with 780 beds, which offers specialised care for patients with rifampicin/multidrug resistant tuberculosis (RR/MDR-TB) and AHD whose problems are too complex to manage at other facilities.

### Sampling strategy

At both hospitals individuals older than 18 years with AHD were identified from medical records. Because the goal of the study was to assess the treatment experiences of participants, individuals with HIV diagnosed in the past 24 months were not included, as we felt their treatment experience would be limited. Among those with AHD hospital clinicians identified those with a CD4 count of < 200 cells/uL and a detectable serum HIV viral load at least twice in the past two years, since it is likely these individuals were experiencing challenges with ART adherence. We had defined sub-optimal adherence as not taking treatment for at least 60 days cumulative in the last two years. Thirty-nine (39) patients were identified by the clinicians. Prior to possible inclusion in the study these 39 patients were approached by the research assistant at their hospital bedside who explained the study and asked about periods in which they may not have been able to take their ART as prescribed in the last two years. Those who had not taken treatment for a least 60 cumulative days (either consecutively or non-consecutively) in the last two years were purposively sampled to ensure diversity in sex, age and period of living with HIV. A total of 21 such individuals were identified and again approached by the research assistant to discuss possible study participation. Our sample was not selected to be fully representative of the entire population of people with AHD but rather to gain insight on the range of experiences of people with AHD in our setting [22]. Our sample size is consistent with other qualitative research in AHD) [23]. On giving consent to participate, participants were given a more detailed explanation of the study, invited to ask questions and then interviewed for the study. Following the interview, they were given a voucher to compensate them for their time.

### Data generation and management

Semi-structured interviews took place between October 1 and November 30, 2023. Twenty-one participants were identified, approached in person and agreed to participate in the study. One patient died before being interviewed, but the remaining 20 (10 at each hospital) were interviewed. Where possible interviews were conducted in a private space, but very ill bed ridden participants who could not be moved were interviewed in the ward. Interviews at King Edward VIII Hospital were interrupted at times by nurses administering medication, but no repeat interviews were done. Interviews lasted between 20 and 90 min, and covered participants’ HIV treatment journeys, their experiences of illness since their HIV diagnosis, their subsequent treatment and care, together with the factors they felt might have impacted their ability to remain in HIV care. The interview guides used at both hospitals were piloted with one participant at each hospital and revised accordingly. In addition to questions on HIV diagnosis and treatment, the guide for King Dinuzulu Hospital included questions on HIV and RR/MDR-TB co-infection and treatment since all participants from this hospital also were being treated for RR/MDR-TB (it is the provincial referral hospital for such individuals). The two guides are attached as Additional file 1 (King Edward VIII Hospital) and Additional file 2 (King Dinuzulu Hospital).

Interviews were done by a female researcher (SH) employed full time by the study, who has 10 years of experience doing open-ended interviews. Interviews were conducted in either English or isiZulu, depending on the preferences of the participant. The interviewer established a relationship with each participant prior to starting the interview by explaining the goals of the interview, her involvement in HIV and TB-related studies for over 15 years, her reasons for being interested in the study and particular interest in understanding the participants’ experience with regards to engaging with HIV services over the course of their illness. Interviews were audio-recorded with consent, transcribed and translated into English for analysis. Transcription and translation were done by a formal research transcription service. Transcripts were not back-translated but reviewed for quality by the interviewer who conducted the interview, and any discrepancies in translation corrected. Feld notes were also taken by the interviewer in case they were needed for clarification, but were not formally analysed. Transcripts were not reviewed with participants and participants did not provide feedback on the findings.

### Data analysis

**Fig.1:**
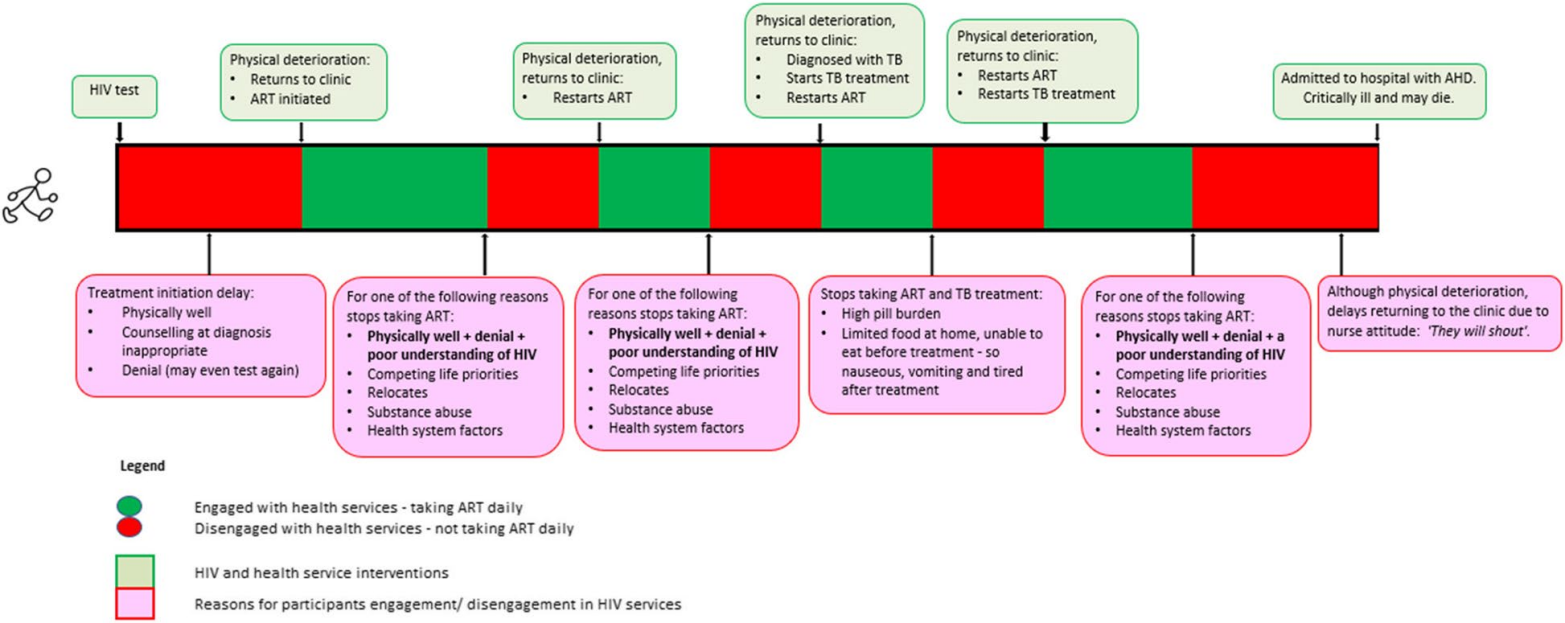
Treatment journey of people hospitalised with advanced HIV disease

Data from transcripts were coded manually and categorised using Excel software. Thematic analysis was done to identify patterns and content [24, 25]. The analysis was guided by two theoretical models. First was the Patient Health Engagement model, which views the person living with HIV as a crucial actor in all phases of the planning and delivery of health care [26]. This framework helped to delineate individual level factors that might be associated with engagement in HIV care. Recognizing the limitations in focusing solely on individual-level factors, our group also employed a second theoretical model called the socio-ecological model. The socio-ecological model views health-related behaviour and decision making as a product of interactions between the individual, the community, and various social institutions [27], and this model was used to contextualize individual experiences within the larger contexts in which they take place. Data analysis was iterative, and transcripts were analysed and reviewed immediately after they became available in English by the study team, with the interview guide updated as needed, to reflect new findings from prior interviews. The initial analytical summaries of the emerging themes and sub-themes conducted by one author (ML) were verified/modified by another author (JF) and discrepancies resolved via discussion until there was agreement among all study team members on the final analytic framework used. A modified version of the coding tree is available in Fig. 1. Thematic data saturation [28, 29] was reached after 17 interviews and recruitment was halted after 20 total interviews when no new codes or themes were identified (determined by ML and JF).

The analysis was also reflexive in nature [30] and identified some potential biases as members of our research team were physicians. This may have led to us overly focusing on biomedical aspects of participants experiences with ART. We tried to adjust for this by elevating the analysis of social experiences in the data interpretation. In addition, as an all-female research team, we recognize our gender may have impacted on the interview process, data analysis, and write-up of the results which could have contributed to marginalization or misunderstanding of male perspectives and voices. We acknowledge these as possible limitations to our analysis and findings.

### Ethics

Written consent was obtained from all patients willing to participate in the study. The consent included information about the study, participation in the interview and digital audio recording, the voluntary terms of involvement in the study, the assurance of confidentiality and anonymity and consent for publication. Anonymity was maintained by identifying each participant by a unique identification number. Ethical approval was obtained from the South African Medical Research Council (SAMRC) Ethics Review Committee (EC050-11/2020) and the KwaZulu-Natal Health Research Committee. This research was carried out and approval and informed consent were obtained in accordance with the World Medical Assembly (WMA) Declaration of Helsinki—Ethical Principles for Medical Research Involving Human Subjects.

## Results

### Participant characteristics

The demographic details of the participants interviewed are summarised in Table 1. The average age was 32 (range 21 – 46) years and equal numbers of males and females were interviewed. Nine participants had been living with HIV for between two and 10 years, the remaining 11 participants had been living with HIV for ≥ 10 years, of whom four had lived with HIV for ≥ 20 years. The route of transmission for most participants (15) was sexual intercourse. Two participants were infected at birth through vertical transmission and a further two when they were raped, one as a 3-year-old and one as a 9-year-old. The remaining participant, an injection drug user, was infected through the sharing of needles. Eighteen of the 20 participants had had at least one episode of TB.

**Table 1:** Demographic and HIV characteristics of participants

Patient ID	Hospital	Gender	Age (years)	Years living with HIV	ART initiation	Probable source of HIV infection
1	KDH^a^	Female	23	16	Diagnosed as living with HIV at 7 years old, ART initiated immediately	Vertical transmission as mother raped
2	KDH	Male	44	9	ART initiated 1 year after diagnosis	Sexual intercourse
3	KDH	Male	35	5	ART initiated at diagnosis	Sexual intercourse
4	KDH	Female	30	5	ART initiated at diagnosis	Sexual intercourse
5	KDH	Male	31	8	ART initiated at diagnosis	Sexual intercourse
6	KDH	Male	33	13	ART initiated 8 years after diagnosis	Sexual intercourse
7	KDH	Male	36	16	ART initiated at diagnosis	Sexual intercourse
8	KDH	Female	39	20	ART initiated a year after diagnosis when CD4 < 200	Sexual intercourse
9	KDH	Female	38	10	2013 diagnosed with HIV	Sexual intercourse
10	KDH	Female	38	12	2011 diagnosed with HIV, but didn’t start Rx immediately	Sexual intercourse
11	KEH^b^	Male	22	22	ART initiated at 9 years old. Participant informed of HIV status at 16 years old	Vertical transmission
12	KEH	Female	26	5	ART initiated at diagnosis	Sexual intercourse
13	KEH	Male	23	2	ART initiated at diagnosis	Sexual intercourse
14	KEH	Male	28	8	ART initiated at diagnosis	Sexual intercourse
15	KEH	Male	36	11	ART initiated at diagnosis	Sexual intercourse
16	KEH	Female	41	10	ART initiated at diagnosis	Sexual intercourse
17	KEH	Male	28	2.5	ART initiated a week after diagnosis	Syringes used for injecting drugs
18	KEH	Female	46	21	ART initiated 18 years after diagnosis	Sexual intercourse
19	KEH	Female	26	21	Diagnosed as living with HIV at 15 years old and ART initiated immediately	Raped as a 3-year-old
20	KEH	Female	22	6	Diagnosed as living with HIV 7 years later and ART initiated immediately	Raped as a 9-year-old

^a^*KDH* King Dinuzulu Hospital

^b^*KEH* King Edward VIII Hospital, *ART* antiretroviral therapy

### Thematic analysis

All participants reported repeated periods of disengagement with the health services. As experiences of periods in which participants disengaged from care was the major finding in this study, our team sought to explore what drove both disengagement from care and re-engagement in care. An overview of some of the factors characterising these periods is provided in Fig. 1 and interview data describing each of these factors is included in the thematic analysis below. Periods of disengagement with services started early, evident both at the time of diagnosis and ART initiation. Disengagement with care continued throughout participants’ treatment journey. Conversely, participants also reported periods of re-engagement with care as well. Excerpts from transcriptions with supporting data are included in the thematic network analysis below.

Three themes were identified as driving the disengagement reported by participants. These were: 1) feeling physically well; 2) life circumstances and relationships; and 3) health systems factors. Because all participants reported periods of time when they re-engaged with care, the fourth theme focused on re-engagement. Re-engagement decisions were often driven by the same overarching factors that drove disengagement, that is feeling physically unwell, life circumstances/relationships, and health systems factors.

#### Disengagement with care theme 1: feeling physically well

Participants’ engagement or disengagement with the health services was driven primarily by how they were feeling physically. If a participant was not experiencing any physical symptoms, s/he reported there was less of an impetus to start or continue HIV treatment and monitoring. At the time of an HIV diagnosis, participants who reported feeling physically well delayed initiating treatment until their health deteriorated:

‘*The thing is…I was also healthy and didn't see a reason for me to take the pills.’ (Participant #6)*

*‘I did not take treatment then (2013), I only started taking treatment in 2021. I just saw that I was alright. Why must I take pills when I am alright?’ (Participant #9)*

Once on ART, an absence of symptoms often combined with a reported lack of understanding of the insidious nature of HIV progression to lead to disengagement in care. Most participants described how when they were feeling physically well ART seemed unnecessary. They reported this led them to disengage from care and ART. These periods of not taking ART are described by some participants below:

*‘I just felt like there was nothing wrong. Like not taking medication and taking medication, it was the same thing.’ (Participant #20)*

*‘I took a risk when I stopped taking the medication, I wanted to test if I would really get very sick like how the nurses said. And I never got sick, I never got sick. (Participant #20)*

*‘Since I didn’t pick up the pills during the weekend, I will pick them up in the following, but that week came and passed, many weeks followed after, because I felt fine, I wasn’t sick. After a couple of years, I started getting sick.’ (Participant #7)*

*‘When I got discharged from hospital I stopped taking the pills because I was starting to feel a bit better. I did not take the pills for a long time.’ (Participant #18)*

One participant took her ART sporadically saying: *‘I did not take my treatment as I felt fine.’* She subsequently developed TB. Below, she describes why she stopped taking both treatments:

*‘It was when I had too many pills to drink……..‘I was not even that ill at that time, I was fine. I could see that I was alright.’ (Participant #9)*

##### Sub-theme: HIV denial or limited understanding of HIV

Feeling physically well sometimes led participants to question or deny their HIV status and/or the need for ART. Many participants cited denial of personal HIV status or the need for daily therapy as a reason for disengaging with care services. This was reported both at the time of initial diagnosis and throughout care. At the time of diagnosis, some participants could not believe their HIV test was positive. This led them to stop going to the facility where they could have been given follow up care and to try “testing again” at another facility. As one participant reported:

*‘I was tested and told that I am HIV positive. They gave me pamphlets that directed me on where I should go since I am positive. I took that pamphlet and threw it inside the bin. After some time I went to another clinic because I was sick. At that clinic you would be asked to take an HIV test irrespective of what brought you there. They tested me and I was given a letter in a white envelope that said I am positive. I started hating clinics.’ (Participant #8)*

*‘They told me at the clinic I was HIV positive and must take pills. But I was well. No-one takes pills when they are well. I didn’t understand I had to take pills even when I was well.’ (Participant #18)*

#### Disengagement with care theme 2: Life circumstances and relationships

Most participants reported that even though they may have been successfully taking ART at some point in their HIV journey, there were things that happened either directly to them (i.e. “life circumstances”) or with their connections to other people in their lives (“relationships”) that had a major impact on their engagement in care. These events and relationships were often seen as directly competing with the health education and messages participants received about HIV and ART in the clinics. But because these were ever-present, and often competing, circumstances and connections that impacted their daily lives, it was challenging to prioritise the benefits of continued ART over them. These two sub-themes are described in more detail below.

##### Sub-theme: the impact of life circumstances

Most participants described how at different times life’s circumstances or more demanding priorities, compounded by limited resources, led to disengaging from care. Three participants described that getting time off work to attend a clinic was, for different reasons, difficult. They needed to work, so they prioritised their jobs and disengaged from care:

*‘I can’t take time off work. If I tell them at work I need to go to the clinic they were going to think “Oh! this boy is sick” and let me go.’ (Participant #11)*

*‘I went to work far from home, and I could not come back on time to fetch my pills. And the nurses get a little rude when you have missed appointments.’ (Participant #14)*

*‘I couldn’t get to the clinic as they (*the bosses*) tell you that production has to keep going, they cannot wait.” (Participant #18)*

In addition to work, other life events could lead to disengaging from care, especially if new responsibilities emerged. For example, one female participant described stopping her treatment after the birth of her child. She reported that she felt well and was too busy coping with her first born to go to the clinic to get her ART and she prioritized caring for her baby over herself now that she felt well:

*‘I was well, but very tired. It was too much to go to the clinic again.’ (Participant #20)*

A participant with no fixed abode described being unable to take his ART when the police confiscated his belongings. Among the personal items of his that they took to stop him living on the street was his ART. He was also unable to take his medication when he did not have water with which to swallow the pills, which he describes in more detail below:

*‘Sometimes I would plan to take the pills before going to sleep but it would happen that I did not have water and I would just sleep……Sometimes you find that the police would confiscate our belongings with the pills inside.’ (Participant #17)*

One participant, a taxi-driver, described how he stopped taking his ART after he was locked out of his house and all his belongings were thrown on the ground. In between trying to recover his possessions, finding accommodation, and continuing to drive his taxi, taking ART and accessing an unfamiliar clinic near his new accommodation could not be priorities. He stated:

*‘I was called by the neighbours, and they said, "Your belongings are being taken out of your home." I stopped working and when I got home, that is exactly what happened. There were new locks. And my sister came. So we took our belongings to where she usually visit her boyfriend, and I was left stranded and didn't know what to do….…There came a time where it's the date to fetch my pills, but where? Its too far to my clinic. I just thought, no, I am stopping this.’ (Participant #2)*

Another participant described how, when food at home was limited, she took her ART without food so that her grandfather could eat. The consequences of this were increased side effects. She described the painful incident below that led to her disengaging from care:

*‘Taking the pills on am empty stomach. They would make my tummy ache, I felt dizzy. I was throwing up, throwing up liquid. I wasn’t even able to concentrate in class and I would sleep……One day when my teacher was hitting me (for sleeping) my bag fell open and my pills got spilled to the floor. I was accused of taking drugs and on the verge of being suspended. So my mother came to explain why I had carried pills to school.’ (Participant #19)*

A couple of participants described how a death or tragedy in the family led to a period of family and personal instability. At times, this could lead to sub-optimal adherence, as described by one participant below:

*‘My partner and I were both on treatment. He then died. I took it* (the treatment*) and stopped, took it and stopped……. And then I stopped completely after my brother died. I was stressed, I lost so many people in my life. My children’s fathers, all are gone.’ (Participant #16)*

##### Sub-theme: the impact of relationships

Relationships were important in either supporting continued engagement in care or facilitating disengagement. Family support was mentioned by several participants as contributing to their ongoing engagement in care. In contrast, feeling alone and unsupported led to disengagement with care, as one participant described below

'*I could not even make conversation with a friend you know and ask: “Friend how were you infected?” That weighed on me a lot and it was tiring that I always have to go to the clinic alone and face those nurses. Those nurses do not have any patience, they do not have time.’ (Participant #19)*

Relationships with friends could be detrimental to ongoing HIV care. Some participants described how engaging in social activities with friends who abused substances and “partied all night” led to poor ART adherence. Two participants described this below:

*‘…..having bad influential friends and not being stable in one place, so I took them (my pills) now and then’ (Participant #1)*

*‘For me it’s always been the alcohol and drinking with my friend. I just did not have time for them pills so I would always say, ‘I will take the pills the next day’ and I would end up saying the same thing even in the next day.’ (Participant #12)*

The fear of inadvertent disclosure, stigma and discrimination continued to impact relationships. Both anticipated and enacted stigma was reported by participants. Stigma limited honesty and open disclosure for several participants. Reflecting this pervasive mistrust, one participant responded when asked if his friends knew about his ART:

*‘There is no such thing as a friend.’ (Participant #11)*

Another participant described a distressing experience when her HIV status was exposed by a school friend. Because of this, she reported her subsequent disengagement from care:

*‘She exposed everything. She literally told them everything. She told them that I have doctors’ appointments every month and they should notice how I don’t show up at school every once a month and they should notice how I am always dressed up on those days. She further said that I go to pick up my pills and they should notice by a jacket I would be wearing. She even told them that I would wear a jacket to hide band aids after I had gotten my bloods taken.’ (Participant #19)*

Not all participants, however, were worried about people finding out they had HIV. Some felt there was an awareness that HIV was common in their community. They reported that this fact gave them less anxiety about disclosure:

*‘With HIV, it is very rare to find out that there are people who do not have HIV. There are people that don't have HIV, but it's no longer something we are wary of.’ (Participant #4)*

One participant described how when he first started ART (around 2010), fearful of disclosure, stigma and discrimination he would not take his ART to parties that lasted all weekend. More recently (2022), as there is more openness about living with HIV he reports knowing which of his friends are on ART. They have decided that together they must take their ART before they start drinking. However, if the party lasts all weekend, they only take their ART on the first day, as he described below:

*‘We would take the pills on the first day we got to our party venue. We usually arrived at these venues around 6pm and we would start drinking around 10pm. So we could make time go into the bathroom, maybe the 6 of us take out the pills from our pockets and drink them around 8pm. We could not take them for the next days.’ (Participant #7)*

##### Sub-theme: the impact of co-morbidities

One life event that had an impact on engagement in care was the development of another health problem. As KDH is the specialised RR/MDR-TB hospital for the province, all participants from this facility had TB. In addition, 8 of the 10 participants interviewed at KEH had had an episode of TB as well (18 of 20 total participants).

One participant who had been living with HIV and on ART for 16 years could not remember how many times he had disengaged from care and not taken his ART. However, he did remember his sub-optimal adherence to both ART and TB treatment during each of his three separate episodes of TB. He, together with several participants (both with drug-susceptible TB and RR/MDR-TB) experienced the side effects of TB treatment as more difficult to tolerate than those of ART. This together with the increased pill burden led to several participants disengaging from health care a couple of months after starting TB treatment, when they started to feel physically well again. They described stopping taking both their TB treatment and ART as outlined below:

*‘The pills annoyed me and I stopped taking them and I just told myself that if I die I will die, I could not stand drinking a lot of pills.’ (Participant #7)*

*‘ART is much easier to take than MDR-TB pills as its only 1 pill and no side effects. MDR-TB pills cause nausea and my skin to darken and peel off.’ (Participant #1)*

#### Disengagement with care theme 3: Health service factors

At the time of their HIV diagnosis a couple of participants described the limitations of the counselling they received at this time. The counselling focussed on the treatment, instead of supporting the person who having just heard of their HIV diagnosis, was in shock. This inevitably led to delays in ART initiation, as some participants reported:

*‘I noticed that I did not undergo counselling that specifically focused on the fact that I just found out that I am HIV positive. So I didn’t have time to process this. I found out and then there was this urgency for me to start treatment because there was no time to waste. I was meant to take the treatment and undergo counselling at the same time. So, when I went through the whole thing, it just went over my head.’ (Participant # 19)*

*‘I was still in my feelings about this whole HIV thing, so I didn’t take the pills seriously……. I do remember there was counselling about the pills, but I don’t remember being told about the side effects. I wasn’t too focused on the counselling. I was not interested because I was still in my feelings.’ (Participant #12)*

Health service “inflexibility” in terms of clinic hours and choice of the clinics they could attend was also described as contributing to disengagement with care, sometimes resulting in delays in ART initiation. One participant described how following his HIV diagnosis in hospital he was told to get his ART from his local clinic. When reflecting on why he did not do this, he described how difficult it is for men to go to clinics:

*‘I got discharged and they advised me that I will start taking the treatment at my local clinic. So, they transferred me to my local clinic. If I can just be honest, men don’t go to clinics…… My local clinic is by our local taxi rank (where taxis queue for passengers), so you get to see everyone there, from people whom you used to go to school with, you’d see people coming from work.…..Most men are scared of the clinic…….. Women go to the clinic for various reasons and do get checkups done, even to get the family planning (contraception) injection. Women go the clinics everyday but us men don’t know how to go to the clinics. It’s hard! Oh, my lord! The first thing is the queuing, men don’t queue. Being on the queue is one thing, but having the whole neighborhood watching you and wondering what illness you have is another story.’ (Participant #7)*

Participants also described a lack of flexibility in how their drugs were advised to be taken as a challenge. Although a few of our participants were on dolutegravir, for which there is some leeway regarding dosing time, all participants described strict medication dosing time recommendations as contributing to sub-optimal ART adherence. The inflexibility of the dosing and services together with the unhelpfulness of clinic staff also contributed to disengagement and in delays in re-engagement following disengagement. One participant described going to a mobile clinic to pick up ART as she did not have the money to take a taxi to the fixed clinic. Mobile clinic staff refused to issue her with medication saying her file was at the fixed clinic. With the help of another patient, she “fooled” the system by pretending she did not know her HIV status. She was immediately offered HIV testing when she said she did not know her status. As a result, she was retested, retested positive and was issued with her ART. She describes her difficulties with the clinic staff at both locations below:

*‘The only issues we have are the clinics…….. If you miss your appointment they shout at you at the clinic, even if you missed your appointment by a day, they still shout at you. They say “this isn’t your appointment date, “and then you wonder how they work because sometimes you find that you still have a few pills left in the container and I explain that I took my pills, it’s not that I did not take them because I would still have a few left. But they still shout at you irrespective. So those are the kind of nurses that we have to deal with.’ (Participant #13)*

Another participant described how, if he was unable to get off work, he might be a day or two late for his appointment. When this happened, the reception he received at the clinic led to him disengaging in care, as described below:

*‘………but the shouting that came from the nurses during that time would make me feel as though I wasn’t human.’ (Participant #7)*

Reported unwillingness of staff and clinics to be accommodating could combine with other life circumstances to drive people out of care. One example of this was when participants reported moving to a new household which required changing clinics. Several described the unhelpfulness of staff as exacerbating their problems of finding a new clinic. In one extreme example, a participant who routinely moved to and from the rural areas (depending on whether he was able to get work in an urban area) ended up disengaged from healthcare because:

*‘They refused to give me a transfer because they cannot give me transfer twice in one year.’ (Participant #3*)

#### Theme 4: re-engagement with care

During the interviewers, it was revealed that the same factors that led to disengagement with care could also lead to re-engagement. Chief among these were a deterioration in physical health. This was described by several participants:

*‘So, every time I had defaulted on treatment my life would be at a standstill because I would get sick, so I was affected in that way.’ (Participant #19)*

*‘I started to feel my body getting weak…..I could feel that a certain percentage of my wellbeing was falling short…..I could tell something was amiss……. Even when I look at myself in the mirror, there would be areas that I notice subtle negative changes, but other people would not notice those changes.’ (Participant #7)*

In terms of re-engagement with care, the diagnosis of a new co-morbidity could also lead to re-engagement. All 18 of the participants who also experienced TB described how prior sub-optimal ART adherence led to the development of their TB symptoms. This spurred their re-engagement with the health services. A taxi driver described his symptoms in the following manner:

*‘I was helplessly sick. I had diarrhoea. I used to sweat a lot at night. I had no appetite. Every time I was waiting at the rank (*where taxis queue for passengers)*, I slept, and I would wake up with a lot of sweat. When a passenger jumps off at a bus stop or if the traffic lights are red, I’d be driving in a lying position. I'd say to myself that I wish I were done working for the day……. When I arrived at the hospital I was admitted. They said I have 'big' TB called MDR.’ (Participant #2)*

Severe illness and hospitalisation were reported to lead to a strengthened commitment to re-engage with healthcare. After being so sick they needed to be hospitalized, many participants vowed to remain in care. Some examples of this are described below:

*‘I’m taking them consistently as I almost died. I know where I come from. There's only a bit left before I wear Pampers (napplies). So I don't dream being on that stage.' (Participant #1)*

*‘I'll end up being lifted from this bed, not being able to walk on my own. These pills are my life.’ (Participant #2)*

*‘I arrived here and I was near death so I don’t see that I will make the same mistake twice. I don’t think that I will be able to endure being sick like that again, because I feel like my ancestors saw me through. So who is going to see me through this time around because ancestors are graceful only for a while? The severity of my illness is something that you might have not been able to endure but I don’t ever wish to experience being sick like that again.’ (Participant #7).*

Participants would start considering whether to re-engage with the health services when they felt poorly, but re-engagement was also impacted by life circumstances/relations and by health services factors. A couple of participants delayed re-engaging due to a lack of money with which to access the health services. Although health services and ART and TB treatment are provided free of charge, costs for transportation or child care may have to be incurred and some patients miss work to attend appointments. However, a more commonly reported reason given was the anticipated rudeness and attitude of the nurses. This not only led to delays in re-engaging with care, but also led to accessing care at another facility:

*‘As I was contemplating going back to the clinic, I started thinking that the nurses will annoy me with their scolding, so I figured I should change clinics and go start taking treatment at a different clinic.’ (Participant #7)*

## Discussion

Despite the increased availability of ART in South Africa, the number of PLHIV hospitalised with AHD has remained stable over the last decade, with increasing numbers of those hospitalised having previously been prescribed ART [31]. This study explored care journeys among people hospitalised with AHD to discern factors that they reported were contributing to their hospitalisation. The most distinct patterns to emerge were the repeated periods of engagement versus disengagement with the health services. Three themes driving disengagement from care emerged: feeling physically well; competing life priorities and relationships; and health services factors. Re-engagement with care was often spurred by feeling physically unwell but was also influence by life circumstances/relationships and by health systems factors.

Feeling physically well was often a reason as to why participants stopped taking ART and disengaged from healthcare, whereas a deterioration in health drove participants to re-engage with healthcare. This pattern was evident both at the time of diagnosis and ART initiation as well as throughout participants’ treatment journey. Studies from southern Africa have reported that PLHIV do not engage with the health services and initiate ART when feeling healthy [32], and a number of studies have reported increased disengagement from care with higher CD4 counts [33–35]. In contrast, it has been reported that PLHIV who have disengaged with care, resume care when their health fails [10, 36, 37]. This dynamic movement in and out of care is ubiquitous [38, 39], and puts PLHIV at risk of poor health outcomes [40, 41]. Participants denial of their HIV status together with a limited understanding of the progression of HIV disease in the absence of signs and symptoms of disease was a sub-theme of feeling physically well and disengaging from care. Other studies have reported that a lack of understanding of the insidious progression of HIV disease contributed to patients believing they did not have HIV and as they were not ill, did not need medical care [33, 34].

Although experiencing physical symptoms was a primary force driving engagement versus disengagement with the health services, the treatment journeys described by our participants were complex given the other factors they had to negotiate and for many participants competing life priorities/relationships emerged as a factor driving disengagement from the health services. Similar findings were reported in a study conducted in the Democratic Republic of the Congo and Kenya which reported that journeys to hospitalisation were marked by social and economic stresses [19]. Others have described the ‘triaging of daily needs’ and ‘competing of survival priorities’ as drivers of disengagement in care, together with reporting that PLHIV re-engage with care when their circumstances change [42–44].

Although there are millions of PLHIV on ART in South Africa [45], we found the fear of discrimination and stigmatisation from inadvertent disclosure of participants HIV status still impacted engagement in care, This fear of disclosure also impacted the formation of intimate relationships, depriving participants of potential sources of support for engagement in care. This fear and its impact on retention in care has been documented in other southern African studies [46, 47]. A limitation of the Universal Test and Treat (UTT) strategy is that at the time of diagnosis the counselling focuses on treatment and not on supporting the person in coming to terms with their HIV diagnosis [48]. Several studies have reported that coping with the trauma of an HIV diagnosis may lead to a delay in ART initiation, motivating some to retest their HIV status at another facility [44, 49].

In addition to negotiating economic and social factors, lack of flexibility in health service location, hours, and medication were reported factors driving delayed engagement at the time of diagnosis/treatment initiation and delayed re-engagement with the health services after a period of disengagement. This is a notable finding in the context of South Africa, where differentiated service delivery (DSD) has been implemented over the past decade [50]. These models of care have been shown to be comparable to traditional models of HIV service delivery in terms of viral suppression and retention in care, but it is noteworthy that as many as 10% of PLHIV using DSD care are no longer taking ART at 24–36 months [51]. Although studies suggest DSD is acceptable for PLHIV overall [52], there are still challenges for some individuals using them, including assignment to a fixed clinic, set “adherence groups” with standard meeting hours, the ongoing need to interact with health care facilities/ providers [53], and concerns about stigma and discrimination in community settings [54].

Because DSD as currently implemented in South Africa still fails to reach a notable and vulnerable group of individuals (who are subsequently at high risk for morbidity, hospitalisation and mortality) in 2021 ‘Operation Phutuma’, a ‘Welcome Back’ strategy was launched [55], aimed at enhancing retention in care, especially for those who had prior periods of disengagement. Instead of experiencing a compassionate return to care when they did decide to re-engage, however, our study participants reported being shouted at by nursing staff. This led many of them to access care at another facility. Moving facilities has been documented as contributing to disengagement with health services [43, 56], and in a Zambian study which followed 544 PLHIV who were no longer in care at their original facility, over half were in care at a new facility [57]. Although a number of participants in our study said they would never disengage from care again, re-engaging with routine outpatients care post-hospitalisation with AHD is often particularly difficult for patients as they may be too ill and weak to travel to a health facility [58, 59]. This period is associated with mortality or readmission and highlights the need for health service providers to engage with patients and develop patient-centred strategies and integrate services across the care-continuum [59].

In our high TB burden setting, most of our study participants were currently sick with TB or had had an episode of TB disease in the past. For a number of these participants, the side effects of TB treatment and increased pill burden led to disengaging from care, and stopping both their ART and TB treatment as soon as they were responding to TB treatment and feeling better [60]. In Table 2 below, recommendations to minimise periods of disengagement with health services and facilitate engagement and reengagement are summarized.

**Table 2:** Recommendations to facilitate ongoing engagement with health services

Recommendations	Details
PLHIV as Partners	Although most healthcare workers In South Africa know about person-centred care, of which the ‘Welcome Back’ strategy is part, our study findings suggest a paternalistic approach to healthcare still exists, where healthcare workers make decisions and, as has been reported elsewhere, the individual needs, values and experiences of PLHIV are overlooked [61, 62]. Patient health engagement models such as the ‘Patients as Partners’ concept have emerged from person-centred care, and gone even further, suggesting **PLHIV should be members of their health care team** as they are key in aligning healthcare decision-making with their daily activities and long-term plans [63]. If PLHIV are goal directed, this will enhance retention in care and lead to improved health outcomes [63–66].
Counselling	**Support at the time of diagnosis:** When someone is newly diagnosed with HIV, providing support as they come to terms with their diagnosis will facilitate engagement with the health services and initiation of ART. **Ongoing support and counselling:** Counselling and peer support can be beneficial in considering HIV status disclosure with family or friends [67]. **Ongoing information:** Continued, and repeated, information on HIV and ART is necessary to address the limited knowledge and understanding of HIV and disease progression. **HIV and TB co-infection:** PLHIV who develop TB need information on the interrelationship between HIV and TB, in addition to counselling and support to cope with the pill burden [68]. Information on possible adverse drug events and returning to the clinic for symptom management can contribute to optimal retention in care. **Grief counselling:** Both HIV and TB involve loss, the loss of personhood, health, partners and family members. In providing person-centred care, identifying PLHIV who are struggling with loss and complicated grief, and providing psychosocial support will enhance retention in care.
Health system factors	Inflexibilities within HIV service delivery need to be addressed including: • the establishment of **efficient person-centred transfer systems** across facilities, • **flexible ART appointment schedules** that accommodate unexpected life events [41], • **multi-month scripting for ART** (3- or 6-months), • **gender sensitive HIV services** to support the retention of men in care [69], • integrating holistic services across the care continuum and the provision of patient-centred services so that very ill and weak patients discharged post AHD can access services, • the provision of ancilliary support at times when life circumstances/relationships are particularly challenging. This could include food, housing and treatment for other illnesses and substance abuse disorders [70].
Ongoing research	Ongoing research and the development and implementation of new technologies such as **long-acting injectable ART** could reduce the frequency of ART visits and minimize competing priorities between HIV care and the stresses of daily life outside of the clinic [39].

*ART* antiretroviral therapy

### Limitations

There are multiple limitations to this study. First, the study relied upon participant recall regarding periods of engaging and disengaging from healthcare, and recall bias may have affected the narratives of these events. Second, some of those we interviewed were very ill and unable to talk at length, which may have limited the information they shared with us. Third, a couple of the participants interviewed at KEH were too ill to move out the ward to a private area to be interviewed. This lack of privacy, together with the busyness of an acute hospital medical ward limited the extent and depth of the interview responses. Fourth, we only included people who had been prescribed ART for 24 months, and thus we may have missed the opportunity to identify factors associated with early disengagement in care. Fifth, this was a small-scale qualitative study whose experiences are unlikely to be generalizable to other populations and further research will be needed to confirm the findings we report here. Sixth, our research team was all female, which may have led to discomfort among male participants and a lack of reporting of their experiences. Finally, our analysis team also included clinical providers which may have led to an over-emphasis on biomedical aspects of care. We minimized this by using non-clinician coders and data analysis team members as well.

## Conclusion

The treatment journeys of people hospitalised with AHD were characterised by periods of engagement and disengagement with the health services. These periods were driven by physical symptoms and competing life priorities/relationships. The changes that occur across a lifetime are not adequately assessed or addressed by current models of service delivery, including DSD. More holistic modes of care—especially those approaches involving ‘PLHIV as Partners’—could be one way to better address changing individual needs across the spectrum of HIV care. Partnership-based service delivery might also encourage supportive relationships that could be called upon to support continued engagement or re-engagement in care. Furthermore, a more balanced power dynamic between service providers and recipients could mitigate the impact of some of the health service factors driving disengagement (i.e. staff attitudes and inflexibility). In addition to re-imagining care as an active partnership, holistic services that focus on life challenges beyond HIV are needed. In the absence of such person-centred initiatives, AHD will likely continue to be a problem for decades to come.

### Supplementary Information

Supplementary Material 1

Supplementary Material 2

## Data Availability

The datasets used and/or analyzed during the current study are available from the corresponding author on reasonable request.
