# Supplementary material for: ‘Not taking medications and taking medication, it was the same thing:’ perspectives of antiretroviral therapy among people hospitalised with advanced HIV disease
Source: BMC Infect Dis. 2024 Aug 13;24:819. doi: 10.1186/s12879-024-09729-8 (PMC11320996; doi:10.1186/s12879-024-09729-8)
Supplement: Supplementary file 1 — Supplementary Material 1 [file 12879_2024_9729_MOESM1_ESM.docx]

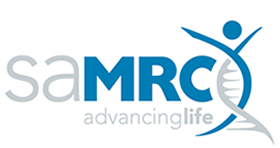


**ADDITIONAL FILE 1: SEMI-STRUCTURED INTERVIEW GUIDE**

**The Treatment Journey of People living with HIV who are hospitalised with Advanced HIV Disease (AHD)**

**_____________________________________________________________________________________**

**Unique study no**: _______________________ **Date:** __­­­___________

**Interviewer:** ___________________________ **Gender: (M or F):** __________

**Date of birth:** _______________________ **Date started ART:** __­­­___________

**Questions:**

1. I believe you are living with HIV? Is this true?
2. When were you first diagnosed with HIV?
3. Can you tell me about the treatment you were given for HIV?

**Probe:** When were you first told to take treatment?

What is your understanding of how you should take the treatment?

What were you told the treatment would do for you?

1. What is it like for you having to take treatment every day?
2. Can you tell me why you are in the hospital?
3. The notes in your medical chart say that you are no longer taking your ART. Can you tell me more about why?
4. Do you think your hospitalisation has any connection to ART?
5. Where it was challenging for you to take your treatment, what made it difficult for you to take your treatment every day?

**Probes:**

- Some people have reported that they have stopped taking their ART when they go to a

traditional healer, faith healer, drink or take drugs. Have you ever been in this situation?

- Some people experience side effects because of ART. Do you experience side effects after taking ART? Are these side effects part of the reason you stopped taking ART?

1. Is this the first time you didn’t take your ART daily?
    If poor adherence has been a problem previously, did stopping treatment make you ill? What made you restart your ART?
2. Can you describe how having HIV and taking ART have affected your life?

**Probes:**

• Does having HIV and taking ART define who you are? (Is being HIV-positive always in the foreground of your mind? Do you ever forget you are HIV-positive? When?)

• Relationships: Have any of your relationships with your close family or friends been

affected by having HIV? What happened?

• Stigma/discrimination? Has anyone or any group in your wider circle of acquaintances

avoided you since you were diagnosed has HIV positive?

• Have you been able to work/provide for your family?

- Many people taking ART gain weight. How do you feel about gaining weight?

1. Have you started taking your treatment again? Do you think you will be able to keep taking

it this time?

**If yes: Probe**: What is going to be different this time?

What will encourage you this time round?

1. Is there anything that could be done to encourage you keep taking your ART?
