# Supplementary material for: ‘Not taking medications and taking medication, it was the same thing:’ perspectives of antiretroviral therapy among people hospitalised with advanced HIV disease
Source: BMC Infect Dis. 2024 Aug 13;24:819. doi: 10.1186/s12879-024-09729-8 (PMC11320996; doi:10.1186/s12879-024-09729-8)
Supplement: Supplementary file 2 — Supplementary Material 2 [file 12879_2024_9729_MOESM2_ESM.docx]

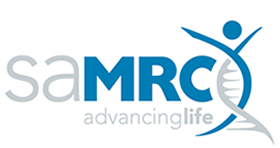


**ADDITIONAL FILE 2: SEMI-STRUCTURED INTERVIEW GUIDE**

**The Treatment Journey of People living with HIV who are hospitalised with Advanced HIV Disease (AHD) and MDR-TB**

**_____________________________________________________________________________________**

**Unique study no**: _______________________ **Date:** __­­­___________

**Interviewer:** ___________________________ **Gender: (M or F):** __________

**Date of birth:** _______________________ **Date started ART:** __­­­___________

**Questions:**

1. I believe you have MDR-TB and HIV. Can you tell me about your HIV?

**Probe:** When were you first told you had HIV and needed to take treatment?

What is your understanding of how you should take the treatment?

What were you told the treatment would do for you?

1. Can you tell me about your MDR-TB?

**Probe:** When were you first told you had MDR-TB and had to take treatment for this?

What is your understanding of how you should take the treatment?

What were you told the treatment would do for you?

1. What is it like for you to take treatment every day?
2. Is there any difference between your ART and MDR-TB treatment?

**Probe:** What is the difference between them?

More pills for MDR? More side effects?

1. Can you tell me why you are in the hospital?
2. The notes in your medical chart say you are no longer taking your medication daily. Are you no longer taking ART or MDR-TB treatment or both?
3. Why are you not taking ART or MDR-TB treatment or both?
4. What has made it difficult for you to take your treatment every day?

**Probes:**

- Some people have reported that they have stopped taking their ART and MDR-TB treatment when they go to a traditional healer, faith healer, drink or take drugs. Have you ever been in this situation?
- Some people experience side effects. Do you experience side effects after taking ART or MDR-TB treatment? Do you think its the ART or MDR-TB treatment that causes the side effects? Are these side effects part of the reason you stopped taking treatment?

1. Is this the first time you didn’t take your ART daily?

If poor adherence has been a problem previously, did stopping treatment make you ill? What made you restart your ART?

10.) Can you describe how having HIV and taking ART have affected your life?

**Probes**

• Does having HIV and taking ART define who you are? (Is being HIV-positive always in the foreground of your mind? Do you ever forget you are HIV-positive? When?)

• Relationships: Have any of your relationships with your close family or friends been

affected by having HIV? What happened?

• Stigma/discrimination? Has anyone or any group in your wider circle of acquaintances

avoided you since you were diagnosed has HIV positive?

• Have you been able to work/provide for your family?

- Many people taking ART gain weight. How do you feel about gaining weight?

1. And what about MDR-TB and taking MDR-TB treatment. How has it affected your life?

**Probes**

• Do you think each day that you have MDR-TB? Do you ever forget you for a whole day or two that you have MDR-TB?

• Relationships: Have any of your relationships with your close family or friends been

affected by having MDR-TB? What happened?

• Stigma/discrimination? Has anyone or any group in your wider circle of acquaintances

avoided you since you were diagnosed with MDR-TB?

• Have you been able to do all the things you were doing before you got MDR-TB?

Things such as housework/looking after children/work/provide for your family?

What has stopped you doing these things? Feeling too ill? Short of breath? Coughing?

12.) Have you started taking your treatment again? Do you think you will be able to keep taking it this

time?

**If yes: Probe**: What is going to be different this time?

What will encourage you this time round?

1. Is there anything that could be done to encourage you keep taking your ART and MDR-TB

treatment?
